# Conservation of direct dynamics in sterically hindered S_N_2/E2 reactions

**DOI:** 10.1039/c7sc04415a

**Published:** 2017-11-13

**Authors:** Eduardo Carrascosa, Jennifer Meyer, Tim Michaelsen, Martin Stei, Roland Wester

**Affiliations:** a Institut für Ionenphysik und Angewandte Physik , Universität Innsbruck , Technikerstraße 25 , 6020 Innsbruck , Austria . Email: roland.wester@uibk.ac.at ; Tel: +43 512 507 52620

## Abstract

Nucleophilic substitution (S_N_2) and base-induced elimination (E2), two indispensable reactions in organic synthesis, are commonly assumed to proceed under stereospecific conditions. Understanding the way in which the reactants pre-orient in these reactions, that is its stereodynamics, is essential in order to achieve a detailed atomistic picture and control over such processes. Using crossed beam velocity map imaging, we study the effect of steric hindrance in reactions of Cl^–^ and CN^–^ with increasingly methylated alkyl iodides by monitoring the product ion energy and scattering angle. For both attacking anions the rebound mechanism, indicative of a direct S_N_2 pathway, is found to contribute to the reaction at high relative collision energies despite being increasingly hindered. An additional forward scattering mechanism, ascribed to a direct E2 reaction, also contributes at these energies. Inspection of the product energy distributions confirms the direct and fast character of both mechanisms as opposed to an indirect reaction mechanism which leads to statistical energy redistribution in the reaction complex. This work demonstrates that nonstatistical dynamics and energetics govern S_N_2 and E2 pathways even in sterically hindered exchange reaction systems.

## Abstract 2

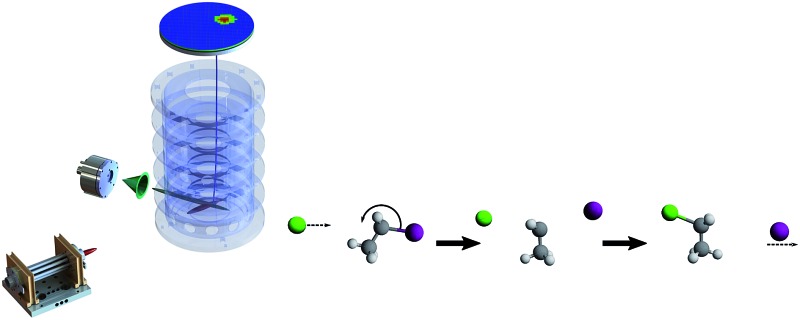
The effect of steric hindrance on the stereodynamics of nucleophilic substitution (S_N_2) and base-induced elimination (E2) has been studied using crossed-beam velocity map imaging.

## Introduction

One of the central characteristics that make bimolecular nucleophilic substitutions (S_N_2) central in organic synthesis is its stereospecific character.^1^ In the simplest approximation, the S_N_2 reaction is assumed to proceed in a collinear fashion, following a three step mechanism: first, the attacking anion approaches the neutral molecule through a long-range attractive ion–dipole interaction. The second step is the formation of an intermediate complex which follows the well known Walden inversion. Finally, the leaving group exits this complex and the final product is formed. The inversion of configuration that occurs during this mechanism is often used in synthetic chemistry to control the formation of specific isomeric compounds.

For many decades significant effort has been devoted to understanding the efficiency of S_N_2 reactions under different conditions and if the proposed collinear attack is universally valid. For that purpose, studies on isolated gas phase reactions appear as an optimal approach. The simplest gas phase nucleophilic substitution reactions between a halide ion and a methyl halide (X^–^ + CH_3_Y) present a minimum energy path resembling a double minimum structure separated by a transition state barrier, which is usually submerged with respect to the reactants' energy.^2^ According to classical statistical models, such as Rice–Ramsperger–Kassel–Marcus (RRKM) theory or transition state theory, the energy available in S_N_2 reactions is randomized among the dense bath of internal degrees of freedom of the intermediate complex through intramolecular vibrational relaxation.^3^ However, early theoretical studies already proposed the occurrence of stereodynamical effects, nonstatistical energy distributions for reaction products and vibrational mode-selective rate enhancement in model X^–^ + CH_3_Y reactions.^4^–^7^ These results were reinforced by experimental studies that demonstrated a different effect of translational and internal energy on the reaction kinetics of such systems.^8^,^9^ Very recent high-level theoretical studies have identified rovibrational mode specificity in model S_N_2 reactions, thus contradicting the predictions of statistical models.^10^–^13^ All these findings have emphasized the importance of carefully considering the timescales for atomic and intramolecular motions along the reaction coordinate in S_N_2 processes.^14^–^16^

Experimentally, crossed beam velocity map imaging studies on X^–^ + CH_3_Y systems have provided evidence of the direct and fast character of S_N_2 reaction pathways such as the direct rebound mechanism, as opposed to a rapid energy randomization.^17^–^19^ In addition, alternative entrance channel geometries such as X–H or X–Y bonded pre-reaction complexes have been identified.^10^,^20^,^21^ For some reactions, such entrance channels have been shown to promote either the formation of new product channels^22^ or to cause a dramatic change in the reaction stereodynamics.^18^,^23^ While the anionic attack on methyl halides (CH_3_Y) can only lead to nucleophilic substitution, reactions with methyl-substituted halides can also follow base-induced elimination, E2. Very recently, we have demonstrated this latter reaction to follow a direct forward scattering mechanism irrespective of the identity of the attacking anion and leaving group.^24^ Complementary theoretical calculations have shown this mechanisms to be a fast process (≈400 fs) that occurs predominantly at high impact parameter collisions.

One key question is if steric substitution may limit the nonstatistical effects observed in X^–^ + CH_3_Y type reactions, that is if energy randomization among the large number of internal degrees of freedom may dominate over reorientation and mode-specific coupling effects in reactions with highly methylated halides.^25^ While many theoretical studies have focused on characterizing the effect of steric substitution on the barrier heights of S_N_2 and E2 reactions,^26^–^29^ computational reaction dynamics studies by means of trajectory calculations are needed in order to investigate the time-dependent steric and rovibrational mode specific effects governing both the branching ratio between S_N_2 and E2 pathways and their intrinsic collision patterns. Studying such factors is even more relevant in systems where nonstatistical effects may govern the reaction outcome, as shown for model nucleophilic substitutions. Further, trajectory calculations can provide important information to laboratory data by assigning experimental scattering angle and velocity ranges to specific reaction products.

To date, computational work on the intrinsic S_N_2/E2 reaction dynamics has been scarce. Only recently, “on the fly” classical dynamics simulations have been performed on F^–^ + CH_3_CH_2_I at one specific collision energy.^24^ While this method does not require computing the full potential energy surface for these systems, it is limited by the long computational time needed to acquire reasonable statistics of reactive trajectories. This can be circumvented by the construction of full-dimensional *ab initio* potential energy surfaces followed by quasiclassical trajectory simulations on them.^18^,^30^ While the complexity of the above-mentioned systems makes the construction of analytical potential energy surfaces demanding, promising progress has been reported very recently.^31^

Experimentally, Bierbaum, Gronert and coworkers have reported a net reaction rate decrease of several X^–^ + RY reactions with increasingly complex neutral reactants, thereby observing a clear relative preference for the E2 pathway as a function of methyl substitution,^32^–^36^ in agreement with our last scattering experiments.^24^ While the studies of Bierbaum and Gronert have provided important structure–energy relations, monitoring the way in which the reactants approach and exchange energy has remained elusive.

The goal of this work is to determine how methyl substitution affects the stereodynamics and energy partitioning in such ion–molecule reactions. We specifically focus on the well-established direct rebound S_N_2 mechanism. For this purpose, we have studied the reactive scattering of Cl^–^ and CN^–^ ions with stepwise methylated alkyl iodides at single collision conditions. Both anions have a low proton affinity and have been shown to disfavour the formation of pre-reaction complexes.^17^,^37^,^38^ Our aim is to investigate the effect of steric hindrance on direct reaction channels.

Table 1 summarizes the S_N_2 and E2 reaction exothermicities and transition state energies for the systems investigated in this work. If compared to the non-substituted Cl^–^ + CH_3_I and CN^–^ + CH_3_I reactions, the S_N_2 mechanisms in Cl^–^ + CH_3_CH_2_I, Cl^–^ + (CH_3_)_2_CHI and CN^–^/NC^–^ + CH_3_CH_2_I also show transition state barriers very near to the reactant energy. In contrast, all *anti*-E2 reactions present positive transition state barriers, although the barrier heights are lower than the lowest relative collision energy studied in the present work (0.4 eV). Thus, all reaction channels presented in Table 1 are energetically accessible given the experimental conditions.

**Table 1:** Reaction enthalpies (Δ_r_*H*) and transition state energies for all considered S_N_2 and E2 reactions (all values given in eV). The exothermicities have been obtained using tabulated formation enthalpies.^39^,^40^ The values indicated in italics correspond to the N-approach of CN^–^. The stationary point energies for the transition state complexes have been calculated at the MP2/aug-cc-pVDZ level of theory

Reaction	Δ_r_*H* (S_N_2)	Δ_r_*H* (E2)	*E*_TS_ (S_N_2)	*E*_TS_ (*anti*-E2)
(1) Cl^–^ + CH_3_I	–0.55	—	–0.08	—
(2) Cl^–^ + CH_3_CH_2_I	–0.67	+0.07	–0.01	+0.28
(3) Cl^–^ + (CH_3_)_2_CHI	–0.68	+0.07	–0.02	+0.10
(4) NC^–^ + CH_3_I	–1.98/*–1.05*	—	–0.21/*–0.03*	—
(5) NC^–^ + CH_3_CH_2_I	–2.00/*–1.16*	–0.65/*+0.01*	–0.05/*+0.10*	+0.14/*+0.29*

We report on a series of gas phase angle- and energy differential cross section measurements of the five reactions listed in Table 1 and show that direct substitution and elimination dynamics persist even when complex neutral partners are involved.

## Results

**Fig. 1:**
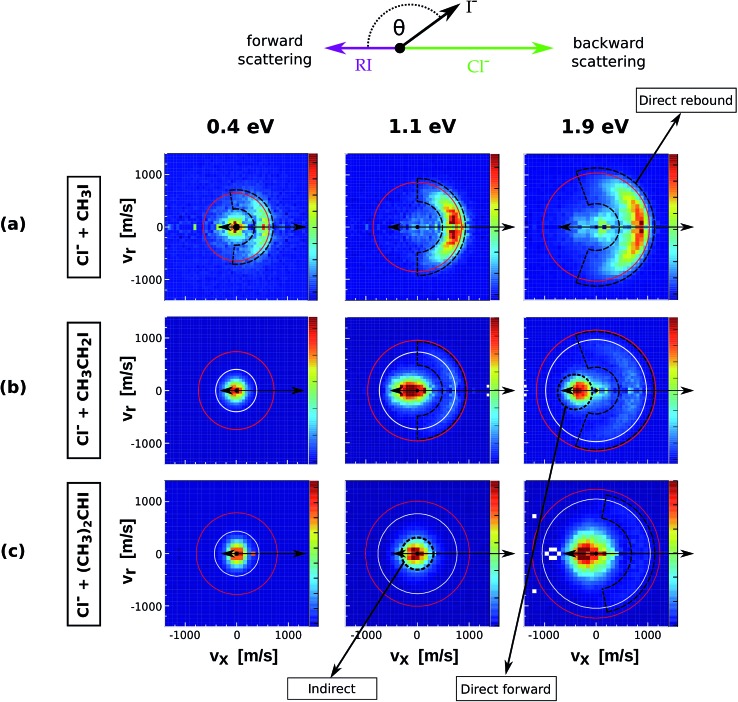
Center of mass velocity distributions of I^–^ ions produced from the reactions Cl^–^ + CH_3_I (a), Cl^–^ + CH_3_CH_2_I (b) and Cl^–^ + (CH_3_)_2_CHI (c). The images present measurements at three different relative collision energies. The arrows depicted above the images illustrate the schematic Newton diagram for the reaction systems, showing the relative direction of the reactant velocities in the center of mass frame and defining forward and backward scattering of the product ion. The red and white rings mark the maximum product ion velocity for the respective S_N_2 and E2 processes, given by energy and momentum conservation. The black dashed regions define the three main mechanistic features that can be distinguished. The data presented for Cl^–^ + CH_3_I correspond to a new set of measurements using an improved version of the instrument compared to the initial study.^17^

**Fig. 2:**
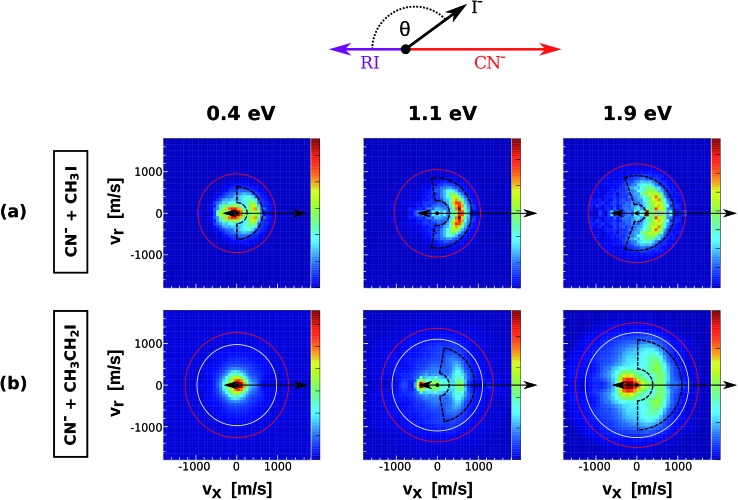
Center of mass I^–^ velocity distributions from the reactions CN^–^ + CH_3_I (a) and CN^–^ + CH_3_CH_2_I (b). The depicted red and white circles mark the kinematic limits for S_N_2 and E2 reactions. The black dashed areas represent the region of direct backward scattered events. The image for CN^–^ + CH_3_I at 1.1 eV has been presented in a recent publication.^38^

The velocity distributions of product I^–^ ions from reactions (1)–(5) (see Table 1) are presented in Fig. 1 and 2. Each velocity image consists of 30 000–40 000 ion events and is depicted in the center of mass frame. The scattering results for the reaction of Cl^–^ with increasingly methylated alkyl iodides are depicted in Fig. 1. The red and white circles drawn in each velocity distribution indicate the kinematic limits for the S_N_2 and E2 reactions, respectively. The kinematic limit is given by the sum of relative collision energy (*E*_rel_) and reaction exothermicity (–Δ_r_*H*, see Table 1).

The upper row presents the velocity distributions of I^–^ ions from the reaction of Cl^–^ + CH_3_I at 0.4 eV, 1.1 eV and 1.9 eV. The velocity map at the lowest *E*_rel_ shows distinct isotropic and backward scattering of the product I^–^ ions relative to the incoming CH_3_I molecules. Isotropic scattering has been associated with the formation of an intermediate complex with a lifetime longer than its rotational period, while backward scattering of the product ion resembles the commonly accepted collinear nucleophilic substitution mechanism.^14^ This latter mechanism strongly dominates at increasing *E*_rel_, with a third distinct feature appearing at 1.9 eV, which has been previously ascribed to a roundabout mechanism.^17^ It is worth noting that only isotropic scattering was observed at *E*_rel_ = 0.4 eV in a previous study on the same system.^17^ The current experimental data recorded at increased velocity resolution are in very good agreement with previous theoretical predictions.^37^

The product velocity images for Cl^–^ + CH_3_CH_2_I (Fig. 1b) present a different dynamical picture. At low *E*_rel_ almost all I^–^ events are scattered isotropically around the center of mass with near-zero velocity. At increasing *E*_rel_ both direct backward and forward scattering features appear. Backward scattered events have a considerably higher velocity than forward scattered events, in many cases appearing above the kinematic limit for E2 products, a fact that reinforces the assignment of these direct backward scattered ions to an S_N_2 mechanism. A considerable fraction of reactions occur *via* a forward scattering mechanism, whose relative abundance increases with the degree of methylation. This scattering pattern has been very recently observed in a series of different reactions with substituted alkyl halides and has been assigned as a fingerprint of E2 reactions.^24^ It cannot be excluded that a fraction of S_N_2 events also occurs *via* forward scattering, as has been shown for similar anion–molecule systems.^20^,^22^ However, forward scattering in S_N_2 reactions has been found to be intimately related to a favoured X–H bonded pre-reaction geometry.^18^ Due to the low proton affinity of both Cl^–^ and CN^–^ and the absence of such a mechanism in Cl^–^ + CH_3_I and CN^–^ + CH_3_I we expect the contribution of forward S_N_2 events to be negligible for the systems discussed here. The forward scattered ions show a very narrow range of product angles at 1.1 eV and 1.9 eV, in contrast to the broad angular distributions of backward scattered events.

For the Cl^–^ + (CH_3_)_2_CHI reaction (Fig. 1c) isotropic scattering dominates at 0.4 eV and 1.1 eV, whereas forward scattering is the predominant feature at 1.9 eV. In contrast to Cl^–^ + CH_3_CH_2_I, backward scattering is only observed at this latter collision energy. Although barely visible in the velocity map, its presence is demonstrated in the corresponding internal energy distribution (Fig. 4, lower panel), where a backward contribution with maximum at ≈1.1 eV can be distinguished. Again, a considerable fraction of these events is scattered with velocities above the kinematic limit for E2, reinforcing the ascription of this mechanism to direct nucleophilic substitution.

Fig. 2 depicts the velocity and scattering angle distributions of I^–^ products from the reactions CN^–^ + CH_3_I (a) and CN^–^ + CH_3_CH_2_I (b) as a function of *E*_rel_. Due to the extremely low reaction rate of CN^–^ + (CH_3_)_2_CHI,^36^ it was not possible to extract statistically meaningful experimental results for this system. The depicted kinematic limits for substitution and elimination correspond to the approach of the C-atom of the CN^–^ ion to the neutral reactant. The scattering features of the velocity images from CN^–^ + CH_3_CH_2_I are very similar to the ones observed in Cl^–^ + CH_3_CH_2_I, with dominant isotropic scattering at low *E*_rel_ and an increase of direct backward scattered events at higher energies. Again, a steric inhibition of this mechanism is observed if compared to CN^–^ + CH_3_I. Forward scattering emerges at 1.1 eV in the same way as in reactions of Cl^–^ with substituted alkyl iodides. Compared to the previous systems, the backward scattered events in CN^–^ + CH_3_I and CN^–^ + CH_3_CH_2_I show lower product velocities, indicating a significant energy partitioning into product internal degrees of freedom.

## Discussion

**Fig. 3:**
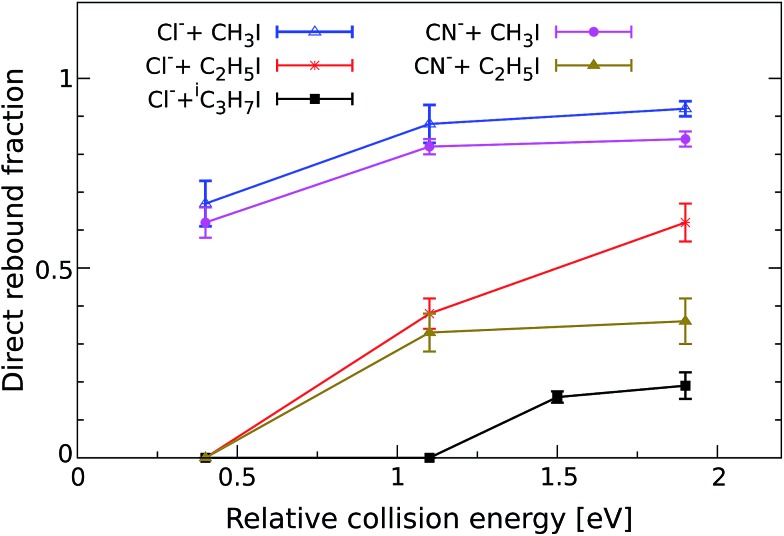
Normalized yield of reactive direct rebound events as a function of *E*_rel_. The yield represents the fraction of total scattering events that fall inside the velocity range of direct rebound scattering, which is marked by the corresponding dashed black area in Fig. 1 and 2. Error bars reflect a convolution of counting statistics and the uncertainties associated with the specific velocity and angle cut. The values for the reaction CN^–^ + CH_3_I have been taken from a recent publication.^38^

In order to quantify the effect of methylation on the relative intensity of the direct backward S_N_2 mechanism, Fig. 3 compares the fraction of direct rebound events for reactions (1)–(5). The fractions have been obtained by defining specific velocity and scattering angle regions for the direct rebound events. These are marked by the black dashed areas shown in Fig. 1 and 2. As the velocity distribution of direct backward events varies depending on reaction system and collision energy, the areas have been defined independently for each velocity image. More details are given in the Methods section.

As a general trend, the fraction of backward scattered events is shown to decrease at lower *E*_rel_ and with increasing steric hindrance at the central carbon. Reactions of both Cl^–^ and CN^–^ with methylated alkyl iodides show no direct rebound at 0.4 eV, whereas the contribution is considerable at this energy in Cl^–^ + CH_3_I and CN^–^ + CH_3_I. Although less predominant than for the non-methylated reactions, backward scattering opens up at 1.1 eV in reactions with CH_3_CH_2_I and at 1.5 eV for Cl^–^ + (CH_3_)_2_CHI, as confirmed by an additional measurement at this energy. Remarkably, more than half of the reactive collisions follow the direct rebound mechanism for Cl^–^ + CH_3_CH_2_I and CN^–^ + CH_3_CH_2_I at 1.9 eV. At the same *E*_rel_ the contribution of this pathway in Cl^–^ + (CH_3_)_2_CHI amounts to 0.15, which is a significant fraction considering the bulkiness of the neutral reactant. It is worth noting that the reported fraction only represents the amount of direct backward S_N_2 events. As shown in Fig. 1 and 2 the forward scattering channel at higher collision energies is also a direct mechanism that has been recently ascribed to an E2 reaction.^24^ Thus, direct scattered products account for the dominating fraction of reactive events at these energies. This demonstrates that direct dynamics still prevail in reactions with highly substituted alkyl iodides.

**Fig. 4:**
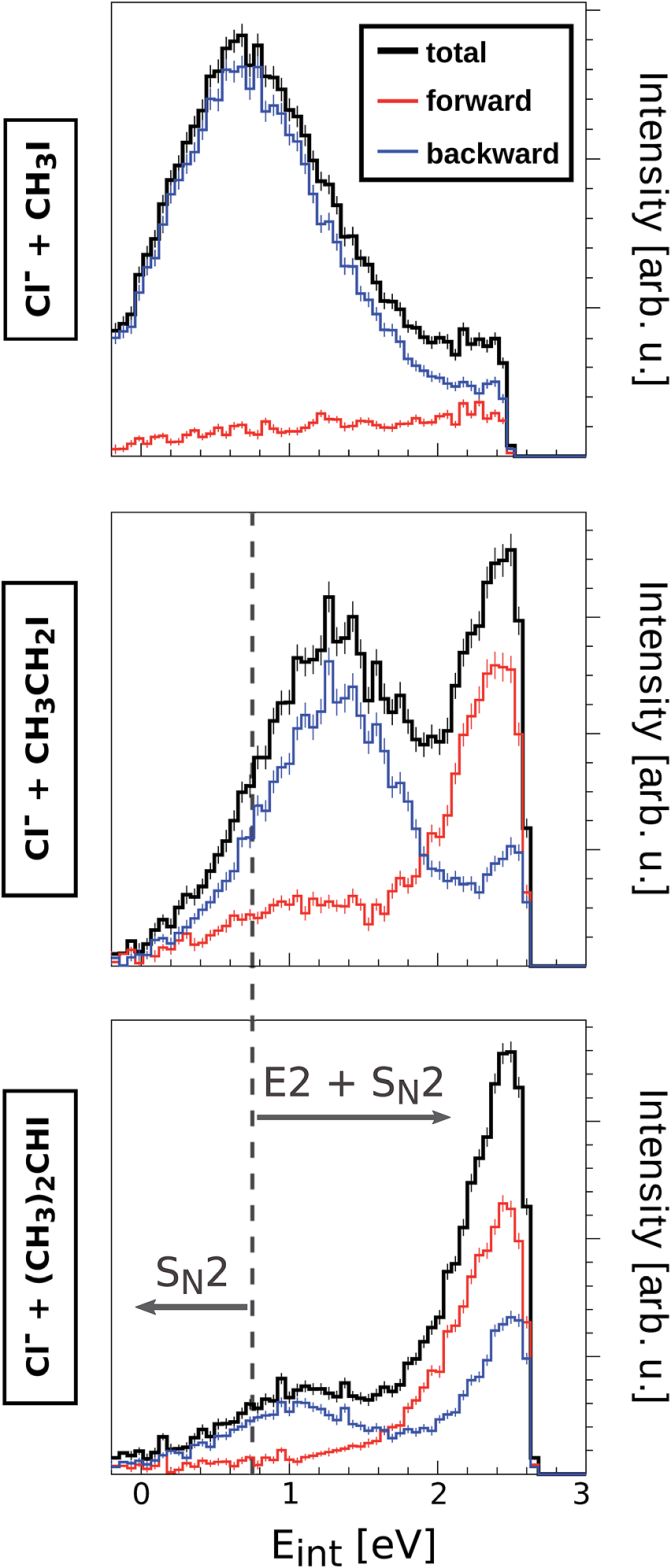
Product internal energy distributions from reactions of Cl^–^ with CH_3_I, CH_3_CH_2_I and (CH_3_)_2_CHI at *E*_rel_ = 1.9 eV (black lines). The red (blue) data show the contribution from scattering into the forward (backward) hemisphere. In all cases, *E*_int_ = 0 corresponds to the kinematic limit for the S_N_2 pathway. The superimposed grey dashed line represents the kinematic limit for the E2 pathway. Product internal energies below this line correspond to S_N_2 collisions.

The direct backward S_N_2 mechanism is known to occur at timescales below 1 ps, *i.e.* considerably faster than intramolecular vibrational redistribution. However, it is important to analyze to what extent steric substitution affects the timescale of the reaction and thus the energy partitioning to the corresponding neutral product. For this purpose, a detailed inspection of the product internal energy distributions (*E*_int_) has been carried out for reactions (1)–(5) at all studied collision energies. Fig. 4 depicts three selected product *E*_int_ distributions from reactions of Cl^–^ with CH_3_I, CH_3_CH_2_I and (CH_3_)_2_CHI at *E*_rel_ = 1.9 eV.

*E*_int_ represents the amount of initially available energy that is partitioned into product internal degrees of freedom. It can be expressed as *E*_int_ = (*E*_rel_ + (–Δ_r_*H*)) – *E*_kin_, with *E*_kin_ being the sum of product ion and neutral translational energies. Long-lived intermediate complexes will favour the initial energy to be redistributed among the product internal degrees of freedom (large *E*_int_), whereas short-lived complexes can result in efficient coupling to product ion translation (small *E*_int_). By extracting the mean of the internal energy for direct rebound events (low *E*_int_ distributions of blue curves in Fig. 4), the corresponding fractions of initial energy partitioned into product internal excitation are obtained for the direct backward S_N_2 mechanism (*f*_*E*_int_(bkw)_, Table 2). The relative error of these fractions is 10–15% and is dominated by the corresponding uncertainty in *E*_rel_.

**Table 2:** Fraction of initially available energy transfered to product internal degrees of freedom in direct backward scattered events, expressed by 
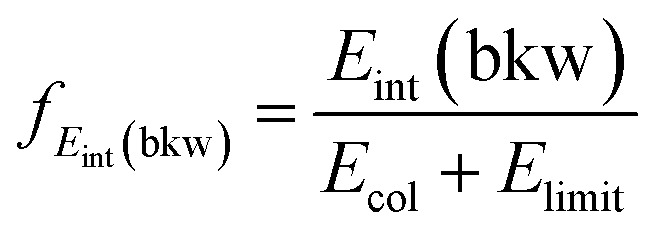
. The relative errors for the fractions are dominated by the uncertainty in *E*_rel_, which amounts to 10–15% in all cases

	0.4 eV	1.1 eV	1.9 eV
Cl^–^ + CH_3_I	0.10	0.20	0.29
Cl^–^ + CH_3_CH_2_I	—	0.41	0.51
Cl^–^ + (CH_3_)_2_CHI	—	—	0.42
CN^–^ + CH_3_I	0.79	0.68	0.69
CN^–^ + CH_3_CH_2_I	—	0.88	0.81

Among the reactions involving Cl^–^, only Cl^–^ + CH_3_I shows direct backward scattering at 0.4 eV. This mechanism is significantly direct, with only 10% of the initial energy being partitioned into internal excitation. At increasing *E*_rel_, the fraction of energy partitioning increases, reaching nearly 30% at 1.9 eV. These data are in good agreement with a measurement performed earlier.^41^ A higher amount of energy is dissipated in form of internal excitation in the reactions Cl^–^ + CH_3_CH_2_I and Cl^–^ + (CH_3_)_2_CHI, however this fraction does not exceed 60% in any case. Strikingly, at high *E*_rel_ direct backward S_N_2 events show slightly less energy partitioning in Cl^–^ + (CH_3_)_2_CHI reactions than in Cl^–^ + CH_3_CH_2_I reactions. This hints to very similar intermediate complex lifetimes and energy transfer in this direct process, irrespective of the degree of methyl substitution. A much higher product internal excitation is observed in reactions with CN^–^, possibly due to the strong coupling of the initial energy to the C–N bond vibration. Similar to the reactions with Cl^–^, the energy partitioning increases from CN^–^ + CH_3_I to CN^–^ + CH_3_CH_2_I. Overall, these results show that the energy partitioning for the direct S_N_2 mechanism at high *E*_rel_ is not significantly affected by adding methyl substituents to the neutral reactant. This finding demonstrates the nonstatistical character of these S_N_2 processes, as fast energy transfer dominates over energy randomization through slow intramolecular vibrational redistribution even in reaction systems with a higher density of states at the intermediate complex than in previously studied reactions.^7^,^8^ Thus, the threshold between nonstatistical and statistical dynamics is not bridged by stepwise methyl substitution but likely has to involve either higher molecular complexity in form of large substituents on the central carbon or a different type of binding interaction.

Given the similarities in reaction dynamics and energetics of the direct S_N_2 channel for reactions (1)–(5), it can be predicted that this mechanism evolves through a collinear entrance channel complex irrespective of the neutral reactant. This would be in consonance with the low proton affinity of both Cl^–^ and CN^–^ ions, and with the observation of a very broad range of scattering angles as indicator of low impact parameter collisions. For such an approach geometry to occur, it is very likely that reactant preorientation takes place and steers the reaction towards the formation of a collinear and short-lived intermediate complex, as predicted for similar systems.^4^,^17^ This is supported by the results presented in Table 2, which show that the energy partitioning does not strongly depend on the substitution pattern. In this regard, the results presented here lay the ground for high level trajectory calculations to be performed in order to add a time-dependent atomistic understanding of the detailed stereodynamics involved in such sterically hindered S_N_2 reactions.

## Conclusion

This work presents experimental reactive scattering results for a series of S_N_2/E2 ion–molecule reactions with stepwise methylated alkyl iodides. We focus on the direct backward S_N_2 mechanism and find that this pathway still occurs at high collision energies in reactions of Cl^–^ and CN^–^ with methylated alkyl iodides. Direct S_N_2 is even favoured both over base-induced elimination and alternative S_N_2 pathways at energies above 1 eV. Analysis of the scattering images and the energy partitioning into internal degrees of freedom shows that both S_N_2 and E2 mechanisms remain direct irrespective of the degree of methyl substitution. The prevailing of such an exchange process is likely related to a stereodynamical effect preorienting the reactants towards a collinear geometry. This study provides the first demonstration of prevailing direct dynamics and nonstatistical energy coupling in S_N_2/E2 reactions with substituted alkyl halides and shows the capability of crossed beam velocity map imaging to study steric effects in complex organic systems.

## Methods

### Experimental procedure

Our experiment combines a crossed beam arrangement with a velocity map imaging (VMI) spectrometer to study the reactive scattering dynamics of the above-mentioned systems at single collision conditions. The whole apparatus operates in pulsed mode at a repetition rate of 20 Hz. The technical details have been reported elsewhere.^42^,^43^ The arrangement consists of three differentially pumped chambers. For the generation of reactant Cl^–^ and CN^–^ ions, low concentration mixtures of CH_3_Cl and CH_3_CN in argon are supersonically expanded into a first chamber through a homebuilt piezoelectric valve and exposed to a discharge between two electrodes. The resulting ions are extracted and mass-selected using a Wiley–McLaren type time-of-flight spectrometer.^44^ Electrostatic lenses and deflectors guide the ions into an octupole radiofrequency ion trap in which a specific ion mass is stored for tenths of milliseconds. During this time the ions undergo non-reactive collisions with nitrogen buffer gas molecules, thereby reducing their kinetic energy spread. The typical full width at half maximum (FWHM) of the ions' kinetic energy lies in the range of 100–150 meV. After extraction the ions are guided and focused into the interaction region of a third chamber containing a velocity map imaging spectrometer. Here, the ions are crossed at a 60° angle with a pulsed supersonic molecular beam, which consists of a low concentration of reactant gas seeded in helium. The neutral gas pulse is generated from a second piezoelectric valve and has a duration of ≈200 μs. The mean velocity of the neutral molecules used in this work is typically in the range of 1200–1300 m s^–1^. The velocity was probed *via* electron impact ionization of the respective neutral beam and subsequent imaging of the CH_3_I^+^/CH_3_CH_2_I^+^/(CH_3_)_2_CHI^+^ product velocity. The measured velocity and angular spreads (FWHM) of the neutral beam are typically 220–240 m s^–1^ and 1.5–2.5 degree, respectively. In order to avoid clustering, the temperature of the neutral beam nozzle is kept at 70 °C and both the backing pressure and reactant concentration of the gas mixture are minimized.

Some of the collisions generate product I^–^ ions, which are extracted normal to the scattering plane by pulsing on the electrodes of the VMI spectrometer. They travel through a drift tube until hitting a microchannel plate, thereby triggering an electron cascade that finally impacts on a phosphor screen. The position and time of the generated light are recorded by a CCD Camera and a photomultiplier, respectively. The accumulated product ion impact positions in the laboratory frame are transformed into a velocity vector in the center of mass frame, resulting in the type of images shown in Fig. 1 and 2. In the case of Cl^–^ the reactive scattering experiments have been performed without discriminating ^35^Cl^–^ and ^37^Cl^–^ prior to the reactive collision. Therefore, 1/4 of the product events are assumed to come from reactions with ^37^Cl. The Newton diagrams have been corrected accordingly in order to resemble the accurate center-of-mass velocity.

In order to obtain the branching ratios for the direct S_N_2 mechanism shown in Fig. 3, appropriate cuts along the product velocity and scattering angle were performed for each velocity image. Thereby, two main assumptions have been made: first, it is assumed that all direct backward scattered events arise from the direct rebound S_N_2 mechanism. Although we have shown that a minor fraction of products from E2 reactions can also follow this scattering pattern in F^–^ + CH_3_CH_2_I,^24^ this should represent a negligible fraction of the total direct backward scattered events. Second, it is assumed that the whole direct backward distribution is inside the cut when changes to the scattering angle or velocity limits do not cause significant changes in the total ion yield inside the range. This is demonstrated below.

**Fig. 5:**
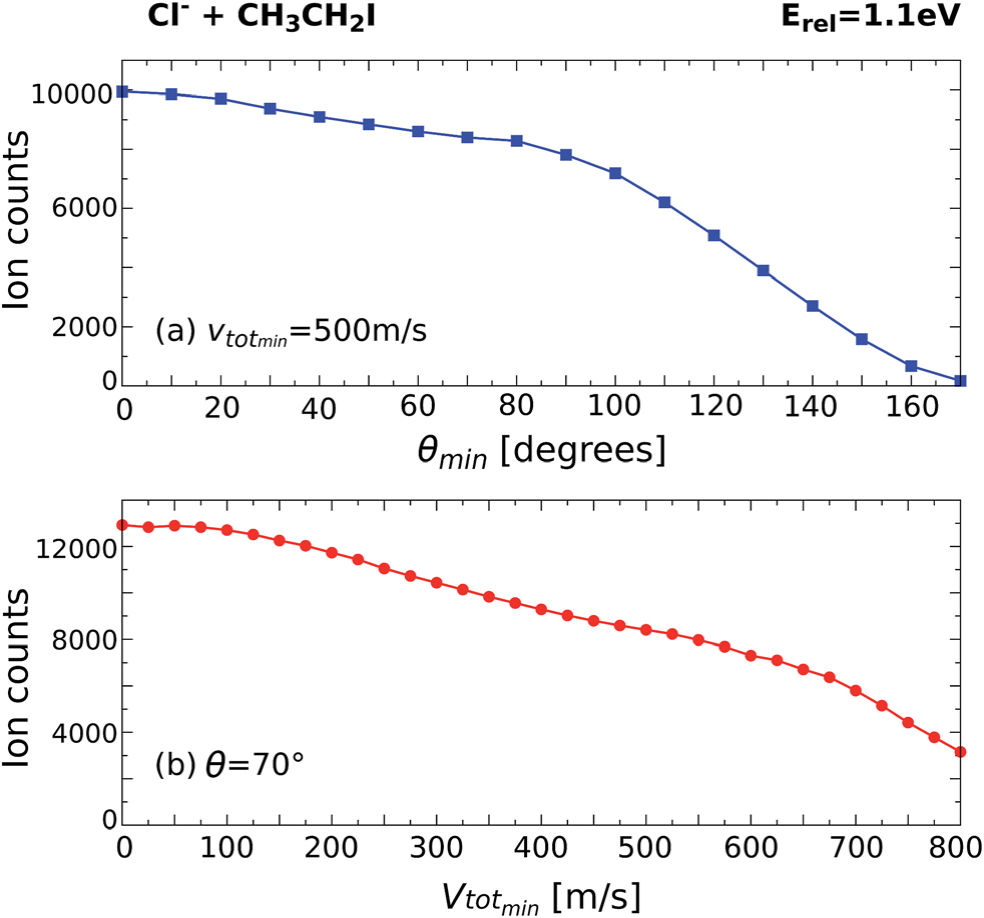
Scans along the product I^–^ scattering angle (a) and minimum total velocity (b) cuts for the reaction Cl^–^ + CH_3_CH_2_I at *E*_rel_ = 1.1 eV.

By monitoring the number of counts as a function of different angle and velocity cuts, general trends can be obtained. Fig. 5 shows example scans along the scattering angle (a) and minimum total velocity (b) of the product I^–^ ion for Cl^–^ + CH_3_CH_2_I at 1.1 eV. Fig. 5a shows the evolution of ion intensity as a function of scattering angle (*θ*) cut for a fixed cut on the minimum total velocity (*v*_tot_min__) of 500 m s^–1^. The cut on the maximum total velocity (*v*_tot_max__) is set to 950 m s^–1^. A steep increase in ion intensity is observed from 170° to 90°, which is the region that contains the backward scattered events. The “plateau” from 90° to 60° marks the outer limit of the scattering angle distribution for the direct S_N_2 mechanism, after which the ion intensity rises again due to the contribution of forward scattered events. Fig. 5b depicts the dependence of ion intensity on *v*_tot_min__ for a fixed scattering angle cut of 70°. We observe a similar steep increase with decreasing *v*_tot_min__, followed by a slight “plateau” from 600 m s^–1^ to 400 m s^–1^. After that, the second increase corresponds to the contribution of the forward scattered events. A second “plateau” below 100 m s^–1^ becomes apparent, indicating that all product events are inside the cut range.

The beginning of the flat regions along the scans define the velocity and scattering angle limits of this mechanism. The range chosen for each image is represented by the black dashed areas in Fig. 1 and 2. The approximate uncertainty of these values is given by *θ* ± 10° for the scattering angle cut and *v*_tot_min__ ± 50 m s^–1^ for the velocity cut. The vertical error bars in the branching ratios of Fig. 3 result from convoluting these two uncertainties.

## Theoretical calculations

The electronic structure calculations have been performed at the MP2/aug-cc-pVDZ level of theory and using the Wadt and Hay relativistic effective core potential (ECP) to represent the iodine core electrons.^45^ All the transition states have been located using the Quadratic Synchronous Transit (QST2) path approximation. The vibrational frequencies were inspected for all considered structures, which allowed the assessment of the zero point energy corrections. All calculations were performed using the Gaussian 09 program package.^46^

## Conflicts of interest

There are no conflicts to declare.
